# Comparison of Survival Outcomes with First-Line PARP Inhibitor Maintenance According to *BRCA* Mutation and Homologous Recombination Deficiency Status in Advanced Ovarian Cancer: A Single-Center Retrospective Cohort Study

**DOI:** 10.3390/cancers18213508

**Published:** 2026-10-31

**Authors:** Young Joo Choi, Jung-Yun Lee, Eun Ji Nam, Sang Wun Kim, Sunghoon Kim, Yong Jae Lee

**Affiliations:** 1 Department of Obstetrics and Gynecology, Institute of Women’s Life Medical Science, Yonsei University College of Medicine, 50-1 Yonsei-ro, Seodaemun-gu, Seoul 03722, Republic of Korea; cyj02097@yuhs.ac (Y.J.C.); shkim70@yuhs.ac (S.K.); 2 Department of Obstetrics and Gynecology, Yonsei University College of Medicine, 50-1 Yonsei-ro, Seodaemun-gu, Seoul 03722, Republic of Korea

**Keywords:** ovarian cancer, *BRCA*, homologous recombination deficiency, PARP inhibitor, olaparib, niraparib, bevacizumab

## Abstract

**Background/Objectives:** First-line poly(ADP-ribose) polymerase inhibitor (PARPi) maintenance is standard in advanced ovarian cancer, but direct comparisons between active regimens within biomarker-defined groups are limited. We compared survival outcomes after first-line PARPi maintenance according to *BRCA* mutation and homologous recombination deficiency (HRD) status. **Methods:** This single-center retrospective cohort included 218 patients who initiated olaparib, olaparib plus bevacizumab, or niraparib after first-line platinum-based chemotherapy. Progression-free survival (PFS) and overall survival (OS) were measured from maintenance initiation. We compared olaparib with niraparib in *BRCA*-mutated disease and olaparib plus bevacizumab with niraparib in HRD-positive, *BRCA*-wild-type disease. **Results:** Overall, 82 patients received olaparib, 33 olaparib plus bevacizumab, and 103 niraparib. Median follow-up was 23.9 months. In *BRCA*-mutated disease, 2-year PFS was 68.2% with olaparib (*n* = 81) and 69.0% with niraparib (*n* = 44; niraparib vs. olaparib: hazard ratio [HR] 1.14, 95% confidence interval [CI] 0.60–2.17; log-rank *p* = 0.695). In HRD-positive, *BRCA*-wild-type disease, 2-year PFS was 81.0% with olaparib plus bevacizumab (*n* = 22) and 62.6% with niraparib (*n* = 51; niraparib vs. combination: HR 1.58, 95% CI 0.61–4.09; log-rank *p* = 0.338). A bevacizumab-containing induction regimen was more frequent in the combination group (13/22 vs. 2/51), and only seven deaths occurred in this comparison. **Conclusions:** Neither comparison showed a statistically significant PFS difference, and the study was not powered to evaluate OS or to establish superiority or equivalence. The small HRD-positive comparison and differences in prior treatment preclude a definitive conclusion about the relative efficacy of olaparib plus bevacizumab and niraparib.

## Simple Summary

After initial chemotherapy for advanced ovarian cancer, many patients receive maintenance tablets called PARP inhibitors to delay the return of the cancer. Which drug is chosen depends on genetic features of the tumor, such as a *BRCA* gene mutation or a related defect in DNA repair. Few studies have directly compared the available options in everyday practice. We reviewed 218 patients treated at one hospital in Korea. In patients with *BRCA* mutations, the time without cancer progression was similar with olaparib and niraparib. In patients with the DNA-repair defect but no *BRCA* mutation, olaparib combined with bevacizumab appeared to delay progression longer than niraparib, but this difference could have occurred by chance because the groups were small and had received different earlier treatments. These real-world results can inform treatment discussions, but larger studies are needed before one option can be preferred.

## 1. Introduction

Ovarian cancer is one of the most lethal gynecologic malignancies [1,2]. Most patients with advanced epithelial ovarian cancer respond to cytoreductive surgery and platinum-based chemotherapy, but recurrence remains common [3,4]. Maintenance therapy aims to prolong disease control after first-line treatment. Approximately half of high-grade serous ovarian carcinomas harbor defects in homologous recombination repair, including germline or somatic *BRCA1/2* mutations [5,6], and such tumors are particularly sensitive to poly(ADP-ribose) polymerase inhibitors (PARPis) through synthetic lethality [7,8]. *BRCA1/2* mutations and homologous recombination deficiency (HRD) therefore help guide the choice of PARPi maintenance therapy [9,10].

Randomized trials have established the benefit of several first-line maintenance approaches. Bevacizumab given with chemotherapy and continued as maintenance therapy prolonged progression-free survival (PFS) in GOG-0218 and ICON7 [11,12]. SOLO1 demonstrated prolonged PFS with olaparib in *BRCA*-mutated disease, with the benefit sustained at 5-year follow-up [13,14]. PRIMA and PRIME evaluated niraparib after first-line chemotherapy [15,16], and veliparib (VELIA) and rucaparib (ATHENA-MONO) have also prolonged PFS in this setting [17,18]. PAOLA-1 demonstrated a benefit from adding olaparib to bevacizumab maintenance after bevacizumab-containing chemotherapy, particularly in HRD-positive disease [19]. Current guidelines recommend selecting first-line maintenance according to *BRCA* mutation and HRD status, together with the use of bevacizumab during chemotherapy [10,20]. Differences in trial populations, induction treatments, and control groups limit comparisons between these active regimens.

The relevant treatment choices also differ by biomarker status. In *BRCA*-mutated disease, olaparib and niraparib are monotherapy options. In HRD-positive, *BRCA*-wild-type disease, niraparib monotherapy and olaparib plus bevacizumab may be considered, depending in part on prior bevacizumab use. The latter comparison therefore involves treatment strategies that can differ before maintenance begins.

Evidence comparing active regimens remains limited. An indirect comparison of SOLO1 and PAOLA-1 examined olaparib with or without bevacizumab in *BRCA*-mutated disease [21], while a Korean multicenter retrospective study compared olaparib and niraparib in *BRCA*-mutated disease [22]. Other Korean real-world series have reported outcomes with olaparib or niraparib maintenance [23,24,25]. More recently, Gasperoni et al. reconstructed individual patient data from PAOLA-1 and PRIMA and found no significant PFS difference between olaparib plus bevacizumab and niraparib in high-risk HRD-positive, *BRCA*-wild-type disease [26]. That analysis, however, relied on cross-trial assumptions about comparable populations and control arms, and real-world data comparing the two regimens in the same population are still scarce for HRD-positive, *BRCA*-wild-type disease.

We examined PFS and overall survival (OS) from maintenance initiation in a single-center cohort. Analyses were conducted separately in *BRCA*-mutated and HRD-positive, *BRCA*-wild-type disease to address the treatment choices relevant to each group.

## 2. Materials and Methods

### 2.1. Study Design and Patients

This single-center retrospective study included 218 patients with International Federation of Gynecology and Obstetrics (FIGO) stage III–IV [27] ovarian, fallopian tube, or primary peritoneal cancer who achieved a complete or partial response to first-line platinum-based chemotherapy and initiated maintenance with olaparib, olaparib plus bevacizumab, or niraparib at Severance Hospital, Seoul, Republic of Korea. Maintenance was initiated between 30 July 2019 and 29 December 2025. The data cutoff was 7 May 2026. The study was approved by the Institutional Review Board of Yonsei University Health System, Severance Hospital (IRB No. 4-2026-0211), which waived the requirement for informed consent.

### 2.2. Treatment and Biomarker Classification

Patients were grouped according to the maintenance regimen initiated: olaparib (*n* = 82), olaparib plus bevacizumab (*n* = 33), or niraparib (*n* = 103). A recorded paclitaxel/carboplatin/bevacizumab regimen was used to identify documented bevacizumab-containing first-line chemotherapy.

*BRCA* mutation and HRD status were obtained from the institutional dataset. HRD was assessed using GreenPlan^®^ HRD (GC Genome, Yongin, Republic of Korea) or whole-genome sequencing (Inocras, Seoul, Republic of Korea). Patients were classified as *BRCA*-mutated (*n* = 135), HRD-positive/*BRCA*-wild-type (*n* = 73), or HRD-negative (*n* = 10). The *BRCA*-mutated analysis compared olaparib with niraparib monotherapy, excluding 10 patients treated with olaparib plus bevacizumab. The HRD-positive, *BRCA*-wild-type analysis compared olaparib plus bevacizumab with niraparib. The HRD-negative group was not included in comparative survival analyses.

*BRCA1/2* status was determined by germline testing in 208 patients and by tumor sequencing in 102, including 92 who had both. Variants were classified according to the ACMG/AMP five-tier system, and only pathogenic or likely pathogenic variants were considered *BRCA* mutations. Patients with only variants of uncertain significance were classified as *BRCA*-wild-type. HRD testing was performed in 103 patients. The assay depended on the treating physician: whole-genome sequencing (Inocras) was used for patients of two physicians (73 patients) and GreenPlan^®^ HRD for the others (30 patients), so each patient was tested with only one assay. For both assays, HRD positivity was defined as a genomic instability score above 42.

### 2.3. Outcomes

The primary endpoint was PFS, measured from the first maintenance dose to documented recurrence or death from any cause, whichever occurred first. Patients without an event were censored at the recorded last follow-up date or the data cutoff (7 May 2026), whichever came first. OS was measured from the first maintenance dose to death from any cause, with surviving patients censored at last follow-up or the data cutoff.

Two-year PFS and OS estimates were reported. Dose reductions, temporary treatment interruptions, and recorded reasons for dose reduction were described separately. Patients could have more than one recorded reason for dose reduction.

### 2.4. Statistical Analysis

Baseline characteristics are presented as numbers and percentages or medians and interquartile ranges (IQRs). Age was compared across the three maintenance groups using the Kruskal–Wallis test, and categorical variables were compared using chi-square tests. Survival analyses were conducted separately in the *BRCA*-mutated and HRD-positive, *BRCA*-wild-type groups. Kaplan–Meier estimates were compared using log-rank tests, and unadjusted Cox models provided hazard ratios (HRs) with 95% confidence intervals (CIs). HRs represent niraparib relative to olaparib in the *BRCA*-mutated comparison and niraparib relative to olaparib plus bevacizumab in the HRD-positive, *BRCA*-wild-type comparison.

Exploratory PFS models were adjusted for FIGO stage IV and absence of gross residual disease. Adjustment was limited because the comparison groups had 39 and 22 PFS events, respectively. All tests were two-sided, and *p*-values were interpreted descriptively.

Median follow-up was estimated using the reverse Kaplan–Meier method. Because the HRD assay depended on the treating physician, we repeated the HRD-positive, *BRCA*-wild-type comparison in patients tested by whole-genome sequencing, the assay used in most of these patients. No sample size was calculated in advance because all eligible patients treated during the study period were included. Based on the observed numbers of PFS events and the Schoenfeld formula, the study had 80% power (two-sided α = 0.05) to detect hazard ratios of about 2.56 (or 0.39) in the *BRCA*-mutated comparison (39 events) and 3.68 (or 0.27) in the HRD-positive, *BRCA*-wild-type comparison (22 events). The analyses were therefore exploratory. They were not designed to test non-inferiority or equivalence, and no margin was set. Statistical analyses were performed using IBM SPSS Statistics (IBM Corp., Armonk, NY, USA).

## 3. Results

### 3.1. Cohort Composition and Baseline Characteristics

The cohort included 82 patients treated with olaparib, 33 with olaparib plus bevacizumab, and 103 with niraparib (Table 1; Figure S1). Median age was 56 years in all three groups, and most patients had high-grade serous histology. FIGO stage IV disease was recorded in 31/82 (37.8%), 23/33 (69.7%), and 52/103 (50.5%) patients, respectively. *BRCA* mutations were present in 81/82 olaparib recipients, 10/33 combination recipients, and 44/103 niraparib recipients. Among patients receiving the combination or niraparib, 22/33 and 51/103, respectively, had HRD-positive, *BRCA*-wild-type disease.

Baseline characteristics within the biomarker-defined comparisons are shown in Table 2. In the *BRCA*-mutated monotherapy comparison, FIGO stage IV disease was recorded in 30/81 olaparib and 27/44 niraparib recipients; no gross residual disease was recorded in 68/81 and 34/44, respectively. In the HRD-positive, *BRCA*-wild-type comparison, FIGO stage IV disease was present in 15/22 combination and 20/51 niraparib recipients. A paclitaxel/carboplatin/bevacizumab induction regimen was recorded in 13/22 (59.1%) and 2/51 (3.9%), respectively. Among the 135 patients with *BRCA* mutations, 113 had germline mutations, and 22 had somatic mutations only.

### 3.2. BRCA-Mutated Monotherapy Comparison

**Figure 1:**
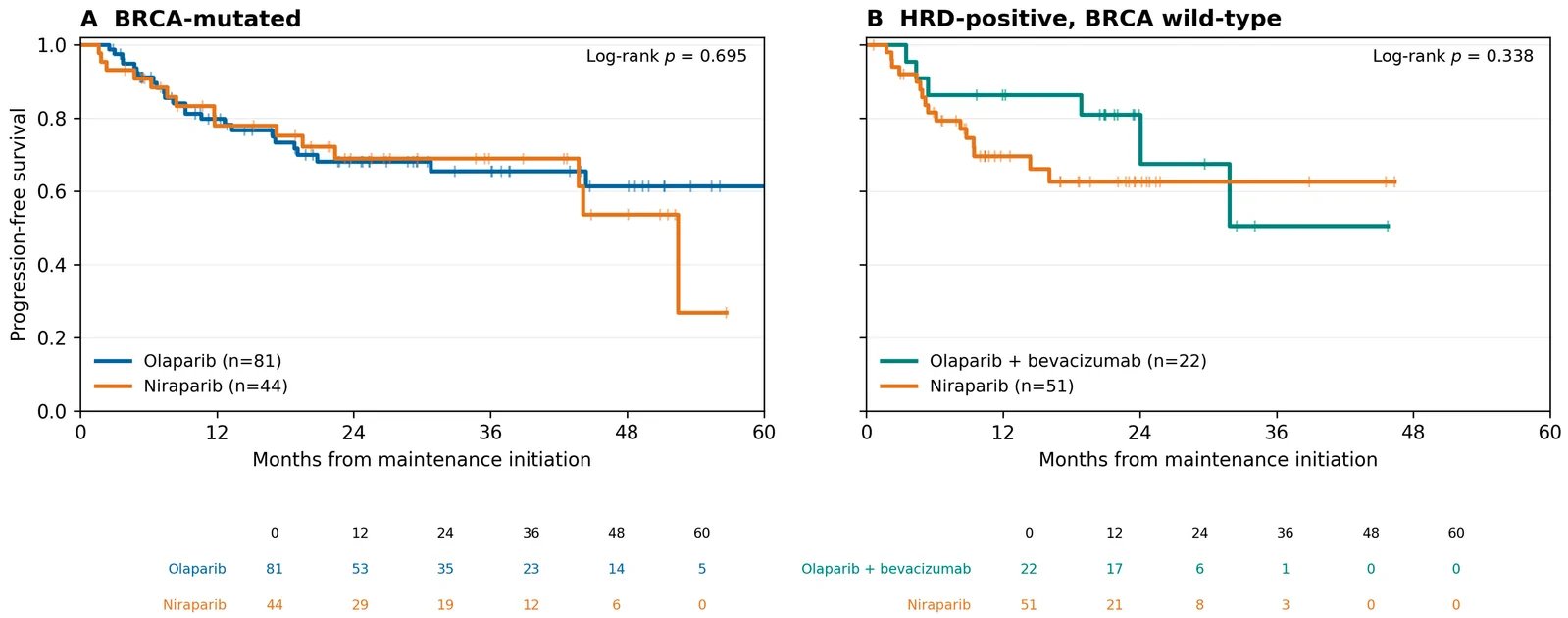
Progression-free survival from maintenance initiation: (**A**) Olaparib (*n* = 81) versus niraparib (*n* = 44) in *BRCA*-mutated disease. (**B**) Olaparib plus bevacizumab (*n* = 22) versus niraparib (*n* = 51) in HRD-positive, *BRCA*-wild-type disease. Kaplan–Meier curves are unadjusted. Tick marks indicate censoring; numbers at risk are shown below each panel. Estimates beyond 30–36 months are based on very few patients at risk, particularly in the olaparib-plus-bevacizumab group, and should be interpreted with caution. HRD, homologous recombination deficiency.

The *BRCA*-mutated comparison included 81 olaparib and 44 niraparib recipients. Ten additional patients with *BRCA* mutations received olaparib plus bevacizumab and were not included in this comparison. PFS events occurred in 24/81 olaparib and 15/44 niraparib recipients. Estimated 2-year PFS was 68.2% and 69.0%, respectively (Figure 1A; Table 3). The unadjusted HR for niraparib versus olaparib was 1.14 (95% CI 0.60–2.17; log-rank *p* = 0.695). After adjustment for FIGO stage IV and absence of gross residual disease, the HR was 0.96 (95% CI 0.49–1.86). Median PFS was not reached with olaparib and was 52.4 months (95% CI 43.7–not reached) with niraparib, although the latter estimate was based on few patients at risk after 48 months. At 24 months, 35 olaparib and 19 niraparib recipients remained at risk for PFS. The median follow-up was 28.7 months (95% CI 24.1–37.7) with olaparib and 34.7 months (95% CI 25.6–42.7) with niraparib.

Eleven olaparib and three niraparib recipients died. Estimated 2-year OS was 88.9% and 94.6%, respectively (Figure S2A; Table 3). The unadjusted HR for niraparib versus olaparib was 0.45 (95% CI 0.13–1.61; log-rank *p* = 0.207).

### 3.3. HRD-Positive, BRCA-Wild-Type Comparison

This comparison included 22 olaparib-plus-bevacizumab and 51 niraparib recipients. PFS events occurred in six and 16 patients, respectively. Estimated 2-year PFS was 81.0% with olaparib plus bevacizumab and 62.6% with niraparib (Figure 1B; Table 3). The unadjusted HR for niraparib versus the combination was 1.58 (95% CI 0.61–4.09; log-rank *p* = 0.338). After adjustment for FIGO stage IV and absence of gross residual disease, the HR was 1.99 (95% CI 0.74–5.34). At 36 months, only one patient in the combination group remained at risk in the PFS analysis. Median PFS was not reached in either group. At 24 months, 6 and 8 patients, respectively, remained at risk. The median follow-up was 23.4 months (95% CI 21.0–45.7) and 17.1 months (95% CI 11.3–23.0), respectively. Whole-genome sequencing was used in 21 of 22 combination recipients and 31 of 51 niraparib recipients; the remaining 1 and 20 patients, respectively, were tested with GreenPlan HRD. When the analysis was restricted to patients tested by whole-genome sequencing (21 combination recipients and 31 niraparib recipients; 20 PFS events), estimated 2-year PFS was 80.0% and 50.1%, respectively (unadjusted HR for niraparib 2.28, 95% CI 0.86–6.00; log-rank *p* = 0.087). After adjustment for FIGO stage IV and absence of gross residual disease, the HR was 3.61 (95% CI 1.24–10.55); however, given the small sample size and limited number of events, this estimate should be interpreted with caution. Niraparib recipients tested with GreenPlan HRD began maintenance later than those tested by whole-genome sequencing and had a shorter median follow-up (11.3 vs. 18.7 months).

Three combination and four niraparib recipients died. Estimated 2-year OS was 94.4% and 92.4%, respectively (Figure S2B; Table 3). The unadjusted HR for niraparib versus the combination was 0.92 (95% CI 0.20–4.17; log-rank *p* = 0.916). The ten patients with HRD-negative disease were not included in either biomarker-defined survival comparison. Median OS was not reached in any group. At 24 months, 44 and 27 patients remained at risk for OS in the *BRCA*-mutated comparison and 8 and 13 in the HRD-positive, *BRCA*-wild-type comparison.

### 3.4. Dose Modifications

Dose reductions were recorded in 37/82 (45.1%) olaparib, 18/33 (54.5%) olaparib-plus-bevacizumab, and 49/103 (47.6%) niraparib recipients (Table S1). Temporary treatment interruptions were recorded in 27/82 (32.9%), 15/33 (45.5%), and 40/103 (38.8%), respectively. Among patients with a dose reduction, recorded reasons included anemia, neutropenia, and thrombocytopenia. Their frequencies by treatment group are provided in Table S1.

## 4. Discussion

This single-center study examined two clinically distinct choices for first-line PARPi maintenance. In *BRCA*-mutated disease, 2-year PFS estimates were close with olaparib and niraparib monotherapy, but the confidence interval was too wide to establish equivalent efficacy. In HRD-positive, *BRCA*-wild-type disease, 2-year PFS was numerically higher with olaparib plus bevacizumab than with niraparib. This comparison was smaller, and the treatment groups differed in their prior therapy and baseline characteristics. Neither finding establishes that one regimen is more effective than the other.

Our *BRCA*-mutated result is broadly consistent with the Korean multicenter retrospective study by Kim and colleagues, which found no significant PFS difference between olaparib and niraparib after propensity score matching [22]. That study also noted that the populations enrolled in SOLO1 and PRIMA differed in characteristics such as stage and timing of surgery [22]. Such differences make the separate randomized trials difficult to use as a basis for choosing between the two drugs. Our analysis directly compared patients within the *BRCA*-mutated stratum, but treatment was not assigned at random, and the number of PFS events was limited. The adjusted estimate remained imprecise. Thus, the absence of a significant difference in our cohort should not be interpreted as proof that olaparib and niraparib have the same effect.

The HRD-positive, *BRCA*-wild-type analysis addresses a different question. Olaparib plus bevacizumab is generally a continuation strategy following bevacizumab-containing chemotherapy, as studied in PAOLA-1, whereas niraparib was evaluated as maintenance after platinum-based chemotherapy in PRIMA and PRIME [15,16,19]. In our cohort, the explicitly recorded paclitaxel/carboplatin/bevacizumab induction regimen was much more common among patients receiving the combination. The comparison therefore encompasses differences that arose before maintenance initiation. Stage IV disease was also more frequent in the combination group. Although we adjusted for stage and residual disease, this limited model could not account adequately for treatment selection or the full history of bevacizumab exposure. This imbalance could not be addressed statistically. A bevacizumab-containing induction regimen was recorded in 13 of 22 patients receiving the combination but in only 2 of 51 receiving niraparib, so the two groups overlapped little in treatment history, and propensity score matching was not feasible. Stage IV disease was also more common in the combination group (15/22 vs. 20/51). Our comparison is therefore closer to one between two treatment pathways (bevacizumab-containing induction followed by olaparib plus bevacizumab and platinum-based chemotherapy followed by niraparib) than between the maintenance drugs alone, and the observed difference may partly reflect how patients were selected for each pathway.

The approximately 18-percentage-point difference in 2-year PFS in this subgroup is clinically interesting, but its uncertainty is substantial. Only 22 patients received olaparib plus bevacizumab, and six experienced a PFS event. The HR confidence interval includes a wide range of possible treatment differences, while few combination-treated patients remained at risk during later follow-up. Vergote and colleagues used a population-adjusted indirect comparison of SOLO1 and PAOLA-1 to examine olaparib with or without bevacizumab in *BRCA*-mutated disease [21]. They emphasized the effects of differences in trial populations, assessment schedules, and prior bevacizumab treatment. Their comparison did not evaluate niraparib or the HRD-positive, *BRCA*-wild-type population and therefore cannot confirm the numerical difference observed here.

Gasperoni et al. likewise found no significant PFS difference between olaparib plus bevacizumab and niraparib in high-risk HRD-positive, *BRCA*-wild-type disease (HR 0.89, 95% CI 0.53–1.49), with restricted mean PFS of 32.4 and 31.2 months, respectively [26]. Their estimate was based on reconstructed trial data and a pooled placebo arm, whereas ours came from patients treated at a single institution. Each approach has its own sources of bias, and neither can show that one regimen is superior. With the number of events in our cohort, only large differences in PFS could have been detected, and OS could not be meaningfully compared.

OS remains particularly difficult to interpret in this cohort. Mature follow-up from SOLO1, PAOLA-1, and PRIMA provides important evidence about the respective regimens against their trial comparators [28,29,30], but it does not directly compare the active treatments examined in our study. In the HRD-positive, *BRCA*-wild-type comparison, only seven deaths occurred. The resulting OS estimate cannot resolve whether the observed PFS difference will be sustained or translate into a survival difference. Subsequent therapy after progression on PARPi maintenance may also affect OS, and in a Korean multicenter cohort the benefit of such therapy differed by regimen [31].

Our time origin was the first maintenance dose. This measures outcomes among patients who responded sufficiently to first-line chemotherapy to begin maintenance and avoids attributing the period before maintenance to a maintenance regimen. Aligning the start of follow-up with treatment initiation is also a key principle for avoiding immortal-time and selection biases in observational comparisons [32]. It does not, however, estimate the effect of choosing one first-line treatment pathway over another at diagnosis. That distinction is especially relevant to the combination group because the decision to use bevacizumab may have preceded maintenance by several months.

Treatment selection also involves tolerability. Kim and colleagues reported differences in hematologic adverse events and dose modifications between olaparib and niraparib [22]. Hematologic toxicity profiles differ among PARPis; thrombocytopenia is more characteristic of niraparib, whereas anemia is common with olaparib [33]. Korean real-world series have described the tolerability of olaparib [23] and the frequent need for niraparib dose modification, mainly for hematologic adverse events [24]. In our cohort, dose reductions and interruptions occurred with all three regimens. Adverse-event grades and permanent discontinuation were not consistently recorded, so these data show only how often doses were reduced or interrupted. They cannot be used to compare toxicity between regimens. This is a meaningful gap, because tolerability often guides the choice among olaparib, niraparib, and olaparib plus bevacizumab in practice.

The study is limited by its retrospective design, single-center setting, small biomarker-defined comparison groups, and potential confounding by indication [34]. Bevacizumab-containing first-line chemotherapy was substantially more common in the olaparib-plus-bevacizumab group, and most patients receiving the triplet regimen had at least four bevacizumab administrations. The observed outcomes therefore reflect differences in treatment pathways as well as maintenance regimens. Two HRD assays based on different methods were used, and their concordance could not be assessed because each patient was tested with only one assay. Because assay choice followed the treating physician, the two assays were unevenly distributed between the treatment groups, and any misclassification of HRD status may have been differential. Such misclassification could bias the comparison in either direction. The PFS difference persisted when the analysis was limited to patients tested by whole-genome sequencing, but this subgroup was small, and the analysis should be regarded as exploratory. With a median follow-up of 23.9 months for the whole cohort and maintenance started as late as December 2025, the PFS data are still immature, particularly for late recurrences. Safety could not be compared between regimens. PFS also depends on the accuracy of recorded recurrence and follow-up dates. Larger studies with balanced treatment histories and longer follow-up are needed to compare these maintenance strategies more reliably.

## 5. Conclusions

No statistically significant PFS difference was observed between olaparib and niraparib in *BRCA*-mutated disease or between olaparib plus bevacizumab and niraparib in HRD-positive, *BRCA*-wild-type disease. The numerical PFS difference in the latter group should be interpreted cautiously because of its small size and differences in prior treatment. The study was not powered to evaluate OS or to show superiority or equivalence of any regimen, and these findings need to be confirmed in larger cohorts.

## Figures and Tables

**Table 1:** Baseline characteristics of the study population (*N* = 218).

Characteristic	Olaparib (*n* = 82)	Ola+Bev (*n* = 33)	Niraparib (*n* = 103)	*p*-Value
Age, years, median (IQR)	56.0 (50.0–63.0)	56.0 (47.0–60.0)	56.0 (49.5–64.0)	0.303
Histology				0.502
High-grade serous	77 (93.9)	32 (97.0)	102 (99.0)	
Other	5 (6.1)	1 (3.0)	1 (1.0)	
FIGO stage				0.007
III	51 (62.2)	10 (30.3)	51 (49.5)	
IV	31 (37.8)	23 (69.7)	52 (50.5)	
Surgery timing				0.204
Primary debulking surgery	47 (57.3)	13 (39.4)	51 (49.5)	
Interval debulking surgery	35 (42.7)	20 (60.6)	52 (50.5)	
No gross residual disease	68 (82.9)	26 (78.8)	81 (78.6)	0.747
Biomarker status				<0.001
*BRCA*-mutated	81 (98.8)	10 (30.3)	44 (42.7)	
HRD-positive/*BRCA*-wild-type	0 (0.0)	22 (66.7)	51 (49.5)	
HRD-negative	1 (1.2)	1 (3.0)	8 (7.8)	
Best response to chemotherapy				0.468
Complete response	70 (85.4)	25 (75.8)	84 (81.6)	
Partial response	12 (14.6)	8 (24.2)	19 (18.4)	

Values are *n* (%) unless otherwise indicated. *p*-Values compare the three treatment groups (Kruskal–Wallis test for age; chi-square test for categorical variables). FIGO, International Federation of Gynecology and Obstetrics; HRD, homologous recombination deficiency; IQR, interquartile range; Ola+Bev, olaparib plus bevacizumab.

**Table 2:** Baseline characteristics within biomarker-defined comparisons.

Characteristic	*BRCA*-Mutated		HRD-Positive/*BRCA*-Wild-Type	
	Olaparib (*n* = 81)	Niraparib (*n* = 44)	Ola+Bev (*n* = 22)	Niraparib (*n* = 51)
Age, years, median (IQR)	56 (50–63)	52 (47.8–59.3)	57 (51.3–61.5)	61 (50.5–67.5)
High-grade serous histology	76 (93.8)	43 (97.7)	21 (95.5)	51 (100.0)
FIGO stage IV	30 (37.0)	27 (61.4)	15 (68.2)	20 (39.2)
Neoadjuvant chemotherapy	35 (43.2)	23 (52.3)	12 (54.5)	24 (47.1)
No gross residual disease	68 (84.0)	34 (77.3)	19 (86.4)	41 (80.4)
Complete response after chemotherapy	69 (85.2)	37 (84.1)	18 (81.8)	42 (82.4)

Values are *n* (%) unless otherwise indicated. FIGO, International Federation of Gynecology and Obstetrics; HRD, homologous recombination deficiency; IQR, interquartile range; Ola+Bev, olaparib plus bevacizumab.

**Table 3:** Survival outcomes from maintenance initiation.

Comparison	Outcome	Events/*N*	2-Year Rate	Unadjusted HR (95% CI)	Log-Rank *p*	Adjusted HR (95% CI)
*BRCA*-mutated: olaparib vs. niraparib	PFS	24/81 vs. 15/44	68.2% vs. 69.0%	1.14 (0.60–2.17)	0.695	0.96 (0.49–1.86)
	OS	11/81 vs. 3/44	88.9% vs. 94.6%	0.45 (0.13–1.61)	0.207	—
HRD-positive/*BRCA*-wild-type: Ola+Bev vs. niraparib	PFS	6/22 vs. 16/51	81.0% vs. 62.6%	1.58 (0.61–4.09)	0.338	1.99 (0.74–5.34)
	OS	3/22 vs. 4/51	94.4% vs. 92.4%	0.92 (0.20–4.17)	0.916	—

PFS and OS were measured from maintenance initiation. HRs compare the second regimen with the first (niraparib as the comparator in both analyses). Adjusted PFS HRs include FIGO stage IV and no gross residual disease. CI, confidence interval; FIGO, International Federation of Gynecology and Obstetrics; HR, hazard ratio; HRD, homologous recombination deficiency; Ola+Bev, olaparib plus bevacizumab; OS, overall survival; PFS, progression-free survival.

## Data Availability

The data presented in this study are available upon reasonable request from the corresponding author. The data are not publicly available due to patient privacy and institutional regulations.

## Supplementary Materials

The following supporting information can be downloaded at https://www.mdpi.com/article/10.3390/cancers18213508/s1, Figure S1: Distribution of 218 patients by first-line maintenance regimen and biomarker status, showing the patients included in each survival comparison; Figure S2: Overall survival from maintenance initiation; Table S1: Recorded dose modifications and reasons for dose reduction.

## Abbreviations

The following abbreviations are used in this manuscript:

CI	confidence interval
FIGO	International Federation of Gynecology and Obstetrics
HR	hazard ratio
HRD	homologous recombination deficiency
IQR	interquartile range
OS	overall survival
PARPi	poly(ADP-ribose) polymerase inhibitor
PFS	progression-free survival
