# Supplementary material for: Comparison of Survival Outcomes with First-Line PARP Inhibitor Maintenance According to BRCA Mutation and Homologous Recombination Deficiency Status in Advanced Ovarian Cancer: A Single-Center Retrospective Cohort Study
Source: Cancers (Basel). 2026 Oct 31;18(21):3508. doi: 10.3390/cancers18213508 (PMC13761458; doi:10.3390/cancers18213508)
Supplement: Supplementary file 1 [file cancers-18-03508-s001.zip › cancers-4650546-supplementary.pdf]

Supplementary Materials

Comparison of Survival Outcomes with First-Line PARP Inhibitor Maintenance According to *BRCA* Mutation and Homologous Recombination Deficiency Status in Advanced Ovarian Cancer: A Single-Center Retrospective Cohort Study

Young Joo Choi, Jung-Yun Lee, Eun Ji Nam, Sang Wun Kim, Sunghoon Kim and Yong Jae Lee

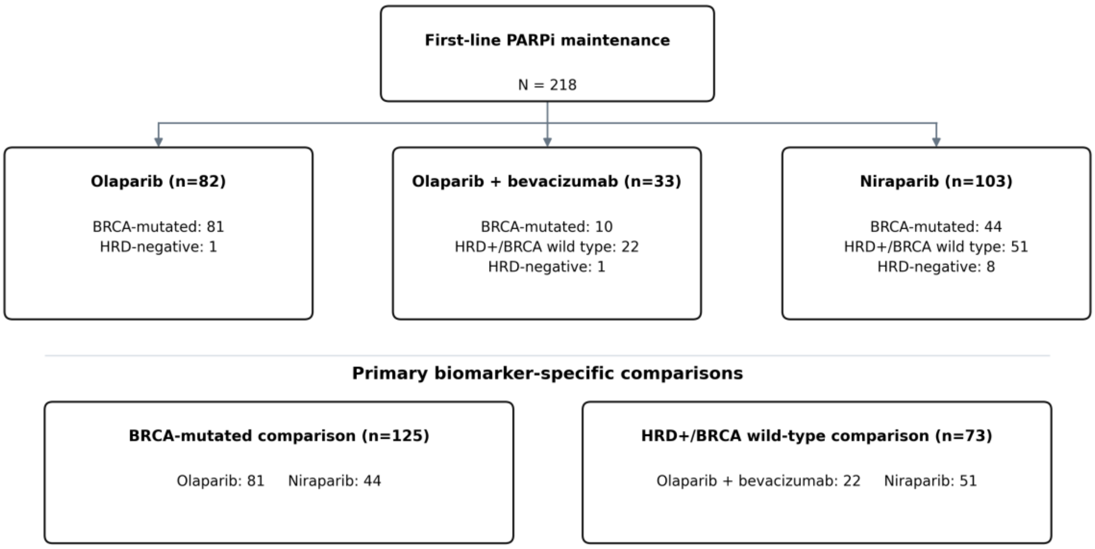

Figure S1. Distribution of 218 patients by first-line maintenance regimen and biomarker status, showing the patients included in each survival comparison. HRD, homologous recombination deficiency; PARPi, poly(ADP-ribose) polymerase inhibitor.

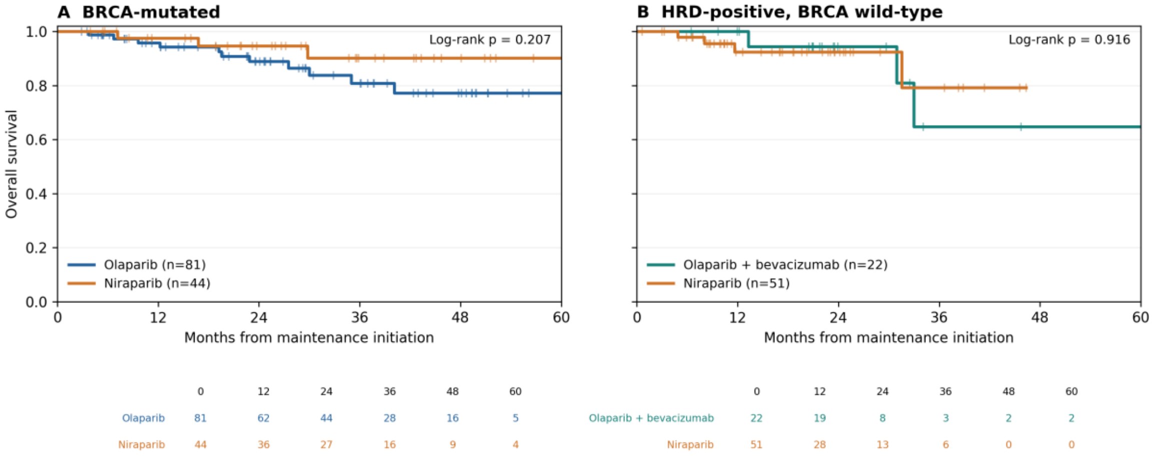

Figure S2. Overall survival from maintenance initiation. (**A**) Olaparib (n = 81) versus niraparib (n = 44) in *BRCA*-mutated disease. (**B**) Olaparib plus bevacizumab (n = 22) versus niraparib (n = 51) in HRD-positive, *BRCA*-wild-type disease. Kaplan–Meier curves are unadjusted. HRD, homologous recombination deficiency. Estimates beyond 30–36 months are based on very few patients at risk and should be interpreted with caution.

Table S1. Recorded dose modifications and reasons for dose reduction.

Characteristic	Olaparib (n = 82)	Ola+Bev (n = 33)	Niraparib (n = 103)
Dose reduction	37 (45.1)	18 (54.5)	49 (47.6)
Dose interruption	27 (32.9)	15 (45.5)	40 (38.8)
Reported reason for dose reduction [a]			
Anemia	9 (11.0)	2 (6.1)	7 (6.8)
Neutropenia	8 (9.8)	5 (15.2)	16 (15.5)
Thrombocytopenia	3 (3.7)	2 (6.1)	10 (9.7)
Nausea	4 (4.9)	0 (0.0)	4 (3.9)
General weakness	4 (4.9)	4 (12.1)	4 (3.9)
Proteinuria	0 (0.0)	1 (3.0)	0 (0.0)

Values are n (%) of each treatment group. [a] More than one reason may be recorded per patient. Niraparib status was unknown for one dose reduction and two interruptions. Ola+Bev, olaparib plus bevacizumab.
